# Microbe Profile: The *Lactobacillaceae*

**DOI:** 10.1099/mic.0.001414

**Published:** 2023-12-13

**Authors:** Jens Walter, Paul W. O'Toole

**Affiliations:** ^1^​ School of Microbiology, University College Cork, Cork, Ireland; ^2^​ APC Microbiome Ireland, University College Cork, Cork, Ireland; ^3^​ School of Medicine, University College Cork, Cork, Ireland

**Keywords:** *Lactobacillaceae*, *Lactobacillus*, *Limosilactobacillus reuteri*, commensalism, probiotic, *Lactobacillus bulgaricus*

## Abstract

The bacterial family *Lactobacillaceae* (the lactobacilli) occupy a unique role in microbiology due to their beneficial role in both human cultural history and biology, from the food preservation of hunter gatherers-turned-farmers, through the prevention of scurvy in seafarers exploring new worlds, and the health-promoting properties of species that colonize the human body as well as animals that are important for agriculture and pollination. The almost bewildering phenotypic and genomic complexity of the former genus *Lactobacillus* was recently reconciled with molecular taxonomy and phylogeny to establish robust genera comprising the *Lactobacillaceae *, whose main features are summarized in this Microbe Profile.

## Graphical abstract

Phylogeny, host association and genome size in the *Lactobacillaceae *. The phylogenetic tree of 287 unique species and 15 strains with unassigned species was built using concatenated alignment of amino acid sequences of core genes, produced by SCARAP (https://journals.asm.org/doi/10.1128/msystems.00264-19) as an input into IQ-TREE2 (https://academic.oup.com/mbe/article/37/5/1530/5721363) with model parameters are described in [2]. Coloured branches represent species divisions established in [2]. Coloured taxon labels indicate major clades and recently established genera. The solid circles by the species names represent genome sizes. The coloured bands on the outer ring indicate the typical habitat based on data in [2].
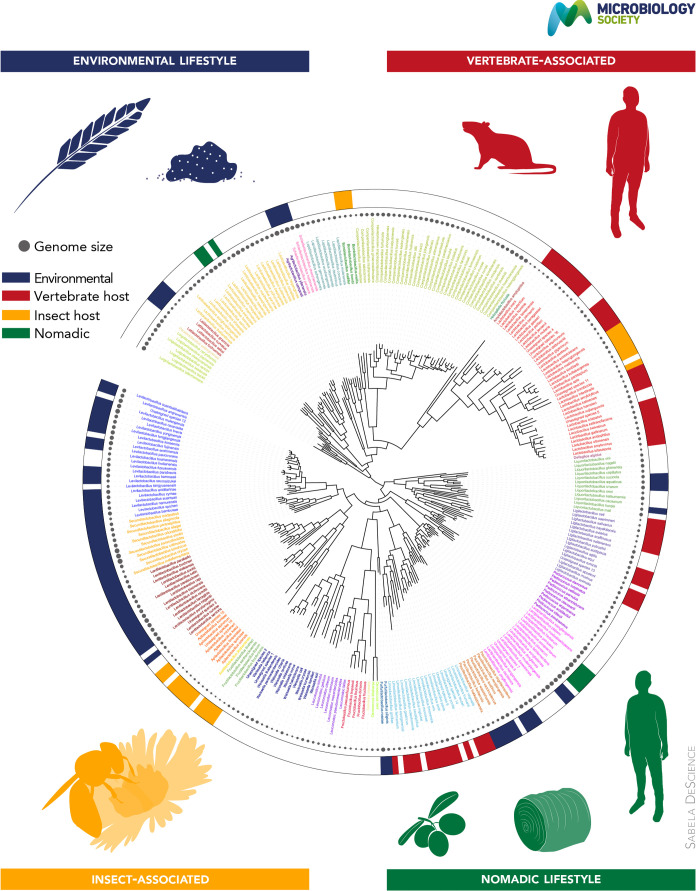

## Taxonomy and phylogeny

Much of what comprises the contemporary family *Lactobacillaceae* was, until 2020, the former genus *Lactobacillus *, which was composed of >260 bacterial species. However, evidence that emerged when genomic sequencing became routine quickly confirmed that the known phenotypic diversity of the then genus *Lactobacillus* was reflected in an extraordinarily high level of genomic diversity that was greater than that of a bacterial family [1]. Based on a polyphasic approach that investigated core genome phylogeny, (conserved) pairwise average amino acid identity and clade-specific signature genes, in the context of physiological criteria and the ecology of the organisms, the former genus *Lactobacillus* was recently subdivided into 25 genera [2]. Most of these genera constitute defined phylogenetic groups that are supported by 100 % bootstrap values (see graphical abstract), indicating that they have been shaped by cohesive evolutionary forces that resulted in clear genomic distinction. These genera, together with the genus *Paralactobacillus *, other genera that were already present before 2020 in the family *Lactobacillaceae* (e.g. *Weisella, Pediococcus* and *Oenococcus *), and the family *Leuconostocaceae *, were united into a single family, the *Lactobacillaceae *, that is now composed of 33 genera and around 300 species.

## Properties and importance

The family *Lactobacillaceae* comprises mostly non-pathogenic (low-virulence) Gram-positive bacteria that inhabit nutrient-rich environments in food, feed, plants, vertebrate and invertebrate animals, and humans (see graphical abstract). They are non-spore-forming facultative or strictly anaerobic bacteria, coccoid or rod-shaped cells that may form pairs or chains [2]. They are nutritionally fastidious (especially those members with smaller genomes) and are grown on complex bacteriological media. *Lactobacillaceae* have substantial economic importance because they are central to the production of food and feed and are also used in biotechnology, bioremediation (compost) and therapeutic applications. Research on this family has therefore been dominated by their role in food fermentations and spoilage, biotechnological applications [1] and their functionality as ‘probiotics’ (‘live micro-organisms, which when administered in adequate amounts confer a health benefit on the host’). These studies have provided important information regarding the metabolism and functionality of the bacteria in a wide array of food environments, fermentations and the gastrointestinal tract of both vertebrates and invertebrates.

The metabolism of the *Lactobacillaceae* is characterized by the fermentation of simple carbohydrates and the production of lactate, acetate and ethanol (the latter two depend on whether species are homo- or heterofermentative, and available substrates). Acid production and the rapid lowering of pH, and the ability to grow competitively under the resulting acidic conditions (and in the presence of salt), are the most important characteristics of fermentation organisms, as this process provides the biochemical conditions necessary for the preservation of food. Acid production is probably also an important protective mechanism for lactobacilli that colonize the vertebrate and invertebrate gut (e.g. in food storage organs such as the crop or forestomach), as well as the human and animal vagina, preventing colonization of these niches by pathogens. However, the metabolism of *Lactobacillaceae* goes beyond simple fermentation and involves a complex range of biochemical processes ranging from lipolysis, proteolysis, bile acid hydrolysis, production of secondary metabolites and other metabolic pathways, as well as the synthesis of bioactive compounds such as vitamins. These processes contribute to food flavour and functionality and impact on the physiology, immunology, fitness and health of humans and animals that are colonized by lactobacilli or consume foods fermented by them. Due to these varied roles, the *Lactobacillaceae* have a unique role in microbiology due to their substantial beneficial impact on social structure, food culture and human history, facilitating the development of human settlements, as well as exploration of the oceans, while keeping humans, and animals important to humans, healthy [3]. Through practices such as back-slopping in the production of fermented foods, and the later development of starter cultures, one could consider the *Lactobacillaceae* the most extensively domesticated bacterial group among all the bacteria.

## Genomics, evolution and ecology

Given their economic importance, most of the research on the *Lactobacillaceae* is dominated by human-made systems (food, feed, biotechnology) and their use as probiotics. As a result, knowledge of the family stems primarily from experimental settings abstracted from any real natural history [4]. It is important to consider that although human-made systems provide opportunities for clonal expansion of strains and local genetic adaptations (often in the form of genome reductions), this evolution is coincidental and recent, and diversification, if it occurs, remains below the species level. From an evolutionary perspective, food, feed and biotechnological fermentations cannot account for the speciation and therefore cannot explain the diversity among the *Lactobacillaceae *. The real ecological context in which the *Lactobacillaceae* exist in nature and how the tremendous diversity within the family evolved, is therefore much less well understood.

The genomic and phylogenomic information that is now available for the wider family and specific species within the family, combined with distribution (isolation) and physiological characterization of isolates, provides insights into the ecology and evolution of the bacterial group in the real world [4]. The genome sizes of the type strains in the former genus *Lactobacillus* were noted to vary remarkably from 1.23 to 4.9 Mb, and with G+C mol% ranging from 31.9–62.8 % [1] (https://www.bacterio.net/family/lactobacillaceae). A core gene family set comprising 114 single-copy core gene families has been identified [2]. Host adaptation in the *Lactobacillaceae* is strongly linked to genome reduction and, interestingly, a reduction in genomic GC content, which constitutes another well-documented general pattern characteristic of genome evolution of host-adapted symbionts [4]. On a broader taxonomic scale, the well-supported phylogenetic groups, which align with new genera, show a remarkable degree of niche conservation (in terms of lifestyle) and can be assigned to either invertebrate and/or flower-associated habitats, different niches within vertebrate hosts (intestine, vagina, oral cavity), free-living (e.g. plants), or to a nomadic lifestyle that transitions between different habitats [2]. There are clear associations of some phylogenetic clades and species with where the corresponding taxa are normally found, as shown in graphical abstract. Overall, the findings are consistent with a model in which free-living ancestors evolved into increasingly host-adapted symbiotics, with present-day species displaying substantial variation in terms of the reliance on environmental niches and the degree of host and niche specificity [4]. On a finer scale, phylogenetic analysis performed for a limited number of species (such as *Limosilactobacillus reuteri *) revealed distinct host adaptations and specialization at sub-species level [5]. The most specialized and strictly host adapted species among the *Lactobacillaceae *, as demonstrated by the smallest genomes (see graphical abstract), are found among the symbionts of insects, as well as in the vaginal species *Lactobacillus iners *.

Lactobacilli can be found in a broad range of hosts and different niches within these hosts, but showing vast differences in their evolutionary histories and ecological roles. In insects, relationships can range from evolutionarily unstable and temporarily dynamic in fruit flies (where highly variable microbiomes are to a large degree shaped by environmental exposure) through to highly stable and specialized associations in bees and bumblebees that clearly qualify as symbioses [4]. *Lactobacillaceae* are also autochthonous (i.e. formed where found) and numerically dominant gut inhabitants in animals such as rodents and birds (including poultry), where some species such as *L. reuteri* form ancient and stable evolutionary relationships with both the host and other members of the microbiota [6]. The evolutionary relationship and ecology of most species of the *Lactobacillaceae* found in the human gut are different. In industrialized countries, lactobacilli are minor members of the human gut microbiota and most species found are actually allochthonous, meaning they originate from food or other sources. Interestingly, in non-industrialized agrarian human populations, the relative proportion of the *Lactobacillaceae* is much higher [7]. The environmental factors that lead to a reduction in the proportional gut abundance of lactobacilli in the industrialized world are unknown but could include reduced availability of fermentable substrates and the lack of transmission of allochthonous microbes through food or contact with animals.

## Health effects

Much research has focused on the consequences of colonization by *Lactobacillaceae* on the health and performance of the host. Most but not all effects are beneficial to the host (the exceptions being rare infections in compromised individuals and oral lactobacilli that have been implicated in dental caries). Among bacterial taxa commercially employed as probiotics, members of the *Lactobacillaceae* are among the most common and the most economically significant, particularly as ingredients of foods with purported health benefits [8]. The mechanisms for such effects have been studied in model systems that range from insects (*Drosophila*, bees) to mammals and through clinical research in humans, and have been established for taxa in the intestinal tract and other host niches (e.g. human vagina). Beneficial effects that have been investigated include reduction of infection risk by providing colonization resistance; improving intestinal barrier function, possibly in conjunction with reducing inflammation; modifying bile acid metabolism and possibly cholesterol metabolism; modulating levels of neurotransmitters involved in mood and behaviour; modulating risk of kidney stone disease by oxalate degradation; acting as a genetically tractable host for biotherapeutic delivery. The fact that most *Lactobacillaceae* are non-pathogenic members of mammalian microbiomes makes them ideal candidates for live microbial therapeutics and probiotics for both humans and animals (agriculture and pets). Although the probiotic literature provides evidence for health benefit of variable quality, there are specific applications for probiotics for which evidence is better and supported through meta-analyses, such as the treatment of different forms of diarrhoea and necrotizing enterocolitis [9].

## Open questions

Although the *Lactobacillaceae* are among the best researched bacterial families with respect to their genome biology, fermentative properties and interesting host associations, important open questions remain about the precise lifestyles, niches, ecology and evolution of species, and the contributions of species to ecosystem services and effects on hosts. Emerging new topics, driven through nutrition trends, include the popularity of fermented foods such as kombucha and kefir, and revived interest in ‘garum’ by Noma, gluten-free sourdoughs in bread production, and plant-based analogues of fermented dairy products or fermented meats. The health effects of fermented foods and the *Lactobacillaceae* therein are enthusiastically described by lay commentators and some scientists but there are few well-designed clinical studies that validate such effects, and the evidence basis for commonly accepted benefits is thin, particularly with respect to the microbiological contribution e.g. for kefir [10]. The clinical validation of the health effects of fermented foods and the underlying mechanisms therefore remains a major area of required research. Interest in the *Lactobacillaceae* and their importance is likely to increase even further with the transition to sustainable food systems to protect planetary and human health. Novel microbial foods based on plant-derived ingredients, novel fermented foods, microbial biomass and food ingredients derived from microbial fermentations are potential solutions for more secure food systems with much smaller environmental footprints than traditional foods. With an outstanding portfolio of safe use, health benefits and metabolic versatility, the *Lactobacillaceae* are key candidates to innovate the food system to achieve sustainable, resilient and healthy human societies.

## Biography

Jens Walter studies the evolution and ecology of gut microbes and the translation of basic microbiome science into therapeutic and nutritional strategies.

Paul O’Toole studies the genomic basis for how gut bacteria impact on health and disease in humans and honeybees.
